# Identification of the Valid Reference Genes for Quantitative RT-PCR in Annual Ryegrass (*Lolium multiflorum*) under Salt Stress

**DOI:** 10.3390/molecules20034833

**Published:** 2015-03-16

**Authors:** Xia Wang, Xiao Ma, Linkai Huang, Xinquan Zhang

**Affiliations:** Grassland Science Department, Sichuan Agriculture University, Chengdu 611130, China; E-Mails: wangxiayuyao@126.com (X.W.); maroar@126.com (X.M.); huanglinkai@sicau.edu.cn (L.H.)

**Keywords:** annual ryegrass, qRT-PCR, reference gene, salt stress

## Abstract

Annual ryegrass (*Lolium multiflorum*) is a cool-season annual grass cultivated worldwide for its high yield and quality. With the areas of saline soil increasing, investigation of the molecular mechanisms of annual ryegrass tolerance under salt stress has become a significant topic. qRT-PCR has been a predominant assay for determination of the gene expression, in which selecting a valid internal reference gene is a crucial step. The objective of present study was to evaluate and identify suitable reference genes for qRT-PCR in annual ryegrass under salt stress. The results calculated by RefFinder indicated that eEF1A(s) was the most stable reference gene in leaves, whereas EF1-a was the least stable; meanwhile, TBP-1 was the most optimal in roots and in all samples, and the eIF-5A shouldn’t be utilized for normalization of the gene expression. eEF1A(s) is more suitable than TBP-1 as reference gene in leaves when verified with P5CS1 and Cyt-Cu/Zn SOD genes. We should choose optimal reference genes in specific tissues instead of the most stable one selected from different conditions and tissues.

## 1. Introduction

Quantitative reverse transcription PCR or quantitative real-time RT-PCR (qRT-PCR) is a well-developed and popular molecular biology technique to rapidly and precisely detect gene expression by measuring relative mRNA levels in cells [1,2,3]. There are two methods to calculate the gene expression in qRT-PCR: absolute quantification and relative quantification. Relative quantification is commonly used to calculate the expression and estimate the levels of the target genes based on housekeeping genes (HKGs) or references genes. Numerous reference genes are used as internal control genes for the Poaceae, such as actin [4], glyceraldehyde 3-phosphate dehydrogenase (GAPDH) [5,6], YT521-B-like protein family protein (YT521-B), eukaryotic elongation factor 1 alpha (eEF1A(s)) [7], and TATA binding protein (TBP-1) [8]. Ideally these HKGs or references genes should be expressed at the same level in different tissues, at all developmental stages or in different experiments. However, numerous recent studies have shown that the reference genes are expressed differently depending the different cells, tissues or experimental conditions [9]. For example, in perennial ryegrass, eIF-4a and 25S rRNA were found to be the most stably expressed genes in roots, whereas EF-1α and UBQ5 were the best in leaf tissues; and eEF-1α and eIF-4a were the most stable when all the tissues when analyzed together [10]. Also for perennial ryegrass, eEF1A (s) and YT521-B were regarded as suitable reference genes with different defoliation management [11]; eIF-4a and TEF1 were considered the most stably expressed genes in drought stress and ABA treatment conditions [12], so eIF4A and TBP-1 should be used as HKGs under salt and heat stress [12]. With the appearance of high throughout transcriptome profiling and microarray technologies, Lin *et al*., have used these technologies to choose the most stable internal control genes from varies reference genes under a series of conditions [13]. Although these technologies helped us find more new reference genes according to the massive amounts of RNA-seq data generated [14], they cannot be universally used nowadays because of the crushing costs. Generally researchers prefer to utilize the traditional method—clone and verify reference genes through the sequence in relative and model plants. Thus we used this method to select valid reference genes depending on different tissues, cells and experimental treatments for accurate and optimal results [15,16,17,18].

Soil salinization is one of the major current eco-environmental problems in agricultural development [19]. At present, humans use fresh water far beyond the biogeochemical cycle capacity and improper agricultural management practices lead to 97.5% water [20] and 6% land area salinization [21]. Nowadays there are about 1 billion hectares of saline soil throughout the globe and this is increasing by some 0.1–0.15 billion hectares per year [22]. Nearly 10% of the saline soil in the world is distributed in China which covers approximately an area of 99 million ha [23]. This situation is more serious along the eastern coastal areas of China and is seriously endangering the sustainable development of agriculture. Annual ryegrass (*Lolium multiflorum*) is cultivated in temperate and subtropical regions worldwide and used in the forage and livestock system as silage and green fodder for its high palatability and digestibility [24,25]. However, saline soils often limit the growth of ryegrass [26], Saline soil is made up with high levels of Na^+^, K^+^, Mg^+^, or Ca^2+^ and Cl^−^, SO_4_^2−^, CO_3_^2−^or HCO_3_^−^, but the major cause is NaCl [27].

Nowadays, there are few reports about the reference genes of annual ryegrass under abiotic stress, so the object of the present work was to identify valid reference genes from the most common ones for qRT-PCR of different tissues of annual ryegrass under salt stress and better normalization of the target genes data, which will be beneficial for taking effective measures to change or to rationally use the saline soil in time.

## 2. Results and Discussion 

### 2.1. Verification of Specificity of RT-PCR and qRT-PCR Products

**Figure 1:**
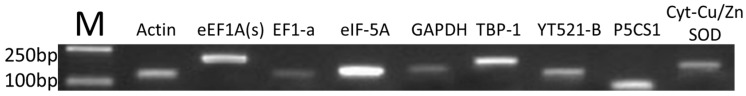
Specific PCR products of nine candidate genes.

**Figure 2:**
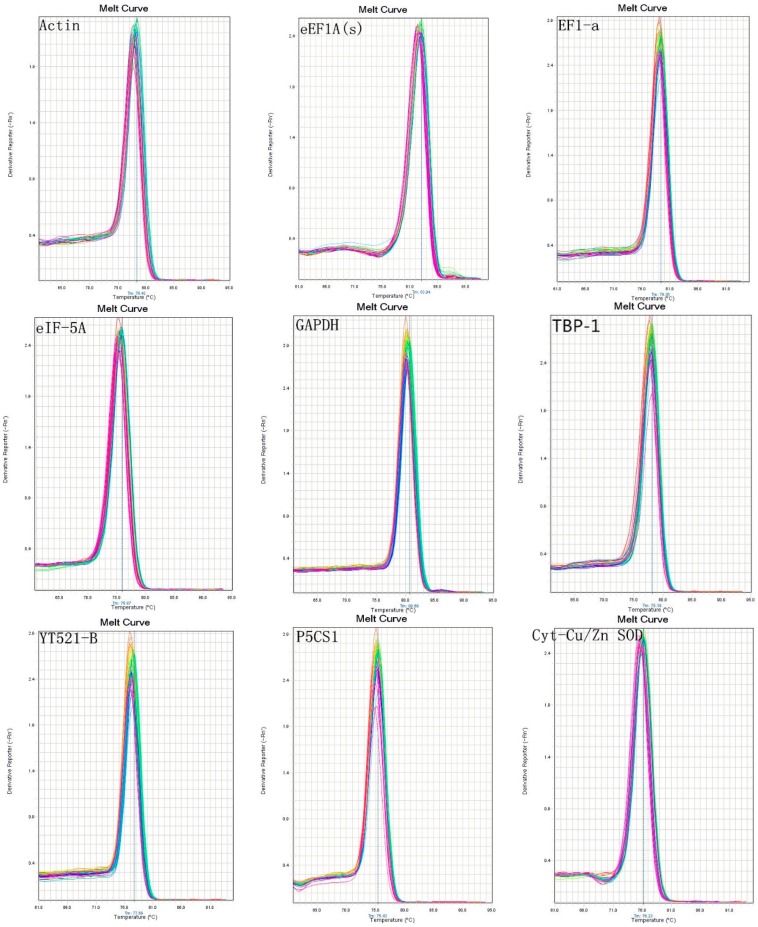
Melt curves of nine genes.

The detailed information of the primers is provided in Table 1. Using RT-PCR to determine the amplicon sizes and specific, Figure 1 showed that there were no primer dimers and no other product amplification. The qRT-PCR products (Figure 2) indicated that there were single-peak melting curves per reference gene.

**Table 1:** Primer sequences of nine genes (seven references genes and two object genes).

Gene Name	Accession ID	Gene Description	Primer Sequence (5'-3')	Amplicon Length (bp)
Actin	AJ585201	actin	F TCCTCACGCCATTCTT	131
			R TCTCCTTGATGTCCCT	
GAPDH	EL664147.1	glyceraldehyde-3-phosphate dehydrogenase	F GCCACCTATGACCAGA	157
			R CGTTCAGAGCAATCCC	
eIF-5A	EL664154.1	translation initiation factor 5A	F CCCCAGGTAAACTTCC	154
			R CAGATAGGTATGGCAAC	
eEF1A(s)	EZ421973	elongation factor 1-α-like protein	F GATGATTCCCACCAAGC	200
			R TAGTAGCAGACAACCACCAG	
EF1-a	Z50789	elongation factor 1-α	F TATTGCCCTGTGGAAGTT	138
			R GTGGTGGAGTCAATGATAAG	
YT521-B	EZ421977	YT521-B-like protein	F AGGGCAAACCAGTCAC	137
			R TTGGCGGTTCTCATAG	
TBP-1	EZ421974	26S protease regulatory subunit-like protein	F CGAGATGCCTTTGAG	188
			R GCGGCAATCACCTTTA	
P5CS1	JX470539	delta-1-pyrroline-5-carboxylate	F ATAACCAATGCTATCCCTGAC R TCTTAGTCGTTGCCTTGA	160
Cyt-Cu/Zn SOD	JQ269677	cytosolic Cu/Zn superoxide	F GGCTGAGTATCCCATTT R CTGCCTTTGCTGTTCT	87

### 2.2. Expression Levels of Seven Reference Genes

**Figure 3:**
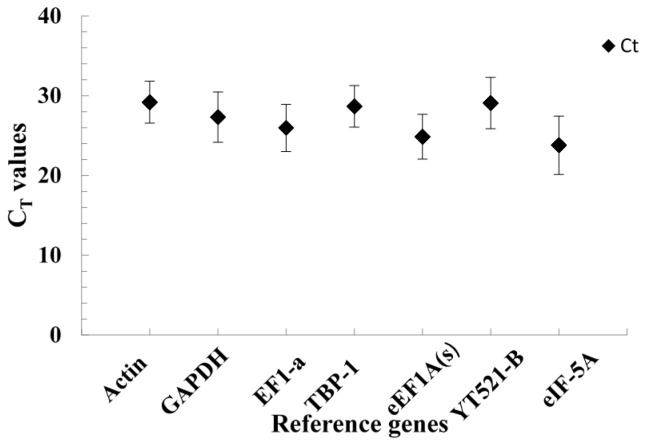
The C_T_ values of seven reference genes in annual ryegrass for all samples (the filled diamond symbol refers to the median C_T_ values of leaves and roots under salt stress, and the bars show the standard deviation).

Figure 3 shows the expression levels of the seven reference genes in all samples by qRT-PCR cycle threshold (C_T_) values. The median C_T_ values of the seven reference genes ranged from 23.79 to 29.19. Especially, the median C_T_ values were around 28 for four reference genes: actin, GAPDH, TBP-1 and YT521-B, which indicated that their expression is not very high. Actin had the lowest expression level. While EF1-a, eEF1A(s) and eIF-5A showed median C_T_ values around 24, and eIF-5A had the highest expression level.

### 2.3. Stability Ranking of Seven Reference Genes in Leaves

RefFinder uses four widely used methods, the comparative ΔCt method, geNorm, NormFinder and BestKeeper. The ranking of the optimal reference genes shown in Table 2 was generated. As for the stability of seven reference genes in leaves, eEF1A(s) and GAPDH were considered to be the best two by the ΔCt, NormFinder and geNorm algorithms, with standard deviation (SD) values of 1.12, 1.15, S values of 0.239, 0.356, and M values of 0.661, respectively. eIF-5A was the least stable in the three algorithms, according to the values stability coefficient (SD = 1.87, S = 1.685 and M = 1.442). Nonetheless, when the BestKeeper method was used, eIF-5A and TBP-1 were the best two with SD values of 0.67 and 1.07, respectively, and EF1-a was the least stable due to its SD value of 2.45. In the overall ranking, eEF1A(s) and GAPDH were the optimum reference genes in leaves under salt stress, while EF1-a was the least stable.

**Table 2:** Evaluation of the expression stabilities of candidate internal reference genes in leaves of annual ryegrass.

Ranking Order (Better—Good—Average) in Leaves							
Method	1	2	3	4	5	6	7
Delta CT	eEF1A(s) 1.12	GAPDH 1.15	Actin 1.23	TBP-1 1.29	YT521-B 1.57	EF1-a 1.86	eIF-5A 1.87
BestKeeper	eIF-5A 0.67	TBP-1 1.07	GAPDH 1.22	eEF1A(s) 1.38	YT521-B 1.46	Actin 1.75	EF1-a 2.45
Normfinder	eEF1A(s) 0.239	GAPDH 0.356	Actin 0.611	TBP-1 0.744	YT521-B 1.188	EF1-a 1.685	eIF-5A 1.685
Genorm	GAPDH | eEF1A(s) 0.661		TBP-1 0.771	Actin 0.871	YT521-B 1.073	EF1-a 1.272	eIF-5A 1.442
Recommended Comprehensive ranking	eEF1A(s) 1.41	GAPDH 1.86	TBP-1 3.13	Actin 3.83	eIF-5A 4.30	YT521-B 5.00	EF1-a 6.24

Data are the stability coefficients of the genes calculated by the software algorithms. The smaller the value, the more stable gene expression is.

### 2.4. Stability of Seven Reference Genes in Roots

The gene expression in roots was lower than in leaves, because the stability coefficients of the reference genes were higher than in leaves (Table 3).

**Table 3:** Evaluation of the expression stabilities of candidate internal reference genes in roots of annual ryegrass.

Ranking Order (Better–Good–Average) in Roots							
Method	1	2	3	4	5	6	7
Delta CT	TBP-1 3.03	Actin 3.18	eEF1A(s) 3.19	EF1-a 3.35	YT521-B 3.41	GAPDH 3.59	eIF-5A 3.99
BestKeeper	YT521-B 2.88	TBP-1 3.42	EF1-a 3.47	Actin 3.63	eIF-5A 3.77	GAPDH 4.11	eEF1A(s) 4.31
Normfinder	TBP-1 1.775	eEF1A(s) 2.048	Actin 2.150	EF1-a 2.302	YT521-B 2.439	GAPDH 2.721	eIF-5A 3.315
Genorm	Actin | TBP-1 1.978		eEF1A(s) 2.597	YT521-B 2.809	EF1-a 2.977	GAPDH 3.156	eIF-5A 3.395
Recommended Comprehensive ranking	TBP-1 1.19	Actin 2.21	YT521-B 3.16	eEF1A(s) 3.35	EF1-a 3.94	GAPDH 6.00	eIF-5A 6.44

The ranking results in roots were as follows: TBP-1 and actin were the most stable two reference genes by the ΔCt and geNorm algorithms, according to their SD values of 3.03 and 3.18, and the M values of 1.978, while eIF-5A was the least stable. YT521-B and TBP-1 were the best by the BestKeeper algorithms on account of their SD values of 2.88 and 3.42, but eEF1A(s) was the least stable with the highest SD value of 4.31. Using the NormFinder algorithms, TBP-1 and eEF1A(s) were suitable reference genes because of their S values of 1.775 and 2.048, and eIF-5A was the least stable (S = 3.315). Based on the comprehensive ranking recommended by RefFinder, the stability values of the TBP-1 and actin were the lowest, thus both of them were the most stable reference genes in roots, while eIF-5A was the least stable.

### 2.5. Stability Ranking of Seven Reference Genes in All Samples

Finally, we combined all C_T_ values to evaluate the expression stability of reference genes (Table 4). In leaves and roots, TBP-1 and actin were the best reference genes by the ΔCt, Normfinder and geNorm algorithms, and eIF-5A was always the least stable. The comprehensive ranking recommended by RefFinder also demonstrated the above conclusion. But when used BestKeeper algorithms, YT521-B and GAPDH were the most optimal, and eIF-5A was still the least stable. Overall, the ultimate results showed that TBP-1 and Actin were the best two reference genes in leaves and roots, while the eIF-5A was the most unstable in this study.

**Table 4:** Evaluation of the expression stabilities of candidate internal reference genes in all samples of annual ryegrass.

Ranking Order (Better–Good–Average) in all Samples							
Method	1	2	3	4	5	6	7
Delta CT	TBP-1 2.60	Actin 2.64	eEF1A(s) 2.80	EF1-a 2.96	GAPDH 3.16	YT521-B 3.22	eIF-5A 3.65
BestKeeper	YT521-B 2.48	GAPDH 2.89	EF1-a 3.34	Actin 3.50	TBP-1 3.55	eEF1A(s) 4.19	eIF-5A 4.34
Normfinder	TBP-1 1.369	Actin 1.540	eEF1A(s) 1.755	EF1-a 2.024	GAPDH 2.386	YT521-B 2.470	eIF-5A 3.101
Genorm	Actin | TBP-1 1.650		eEF1A(s) 2.020	EF1-a 2.355	GAPDH 2.607	YT521-B 2.747	eIF-5A 3.005
Recommended Comprehensive ranking	TBP-1 1.50	Actin 2.00	eEF1A(s) 3.57	EF1-a 3.72	YT521-B 3.83	GAPDH 3.98	eIF-5A 7.00

### 2.6. Validation of the Stability Reference Genes Identified from This Study

In order to identify which reference gene was the most optimal, we analyzed two salt stress-related genes: delta-1-pyrroline-5-carboxylate (P5CS1) and Cyt-Cu/Zn superoxide dismutase (Cyt-Cu/Zn SOD). In plants, P5CS1 gene is the key enzyme in the proline biosynthesis pathway [28]. Proline acts as an osmoprotectant and protein stabilization is very important in the response to salt stress [29]. Overexpression of the P5CS1 gene can increase the concentration of proline to resist the salt stress [30,31,32,33,34]. SOD provides the first line of defense against oxidative stress which has been proposed to be important in salt tolerance. SODs are classified in four types based on their different cellular compartments: the copper/zinc type (Cu/Zn SOD); the manganese (Mn SOD); the iron type (Fe SOD) and the nickel type (Ni SOD). The Cu/Zn SOD is located in the chloroplast and cytosol, so it is further divided into Chl-Cu/Zn SOD and Cyt-Cu/Zn SOD [35,36]. Cyt-Cu/Zn SOD is up-regulated in salt stress to avoid the effect of ROS [37].

**Figure 4:**
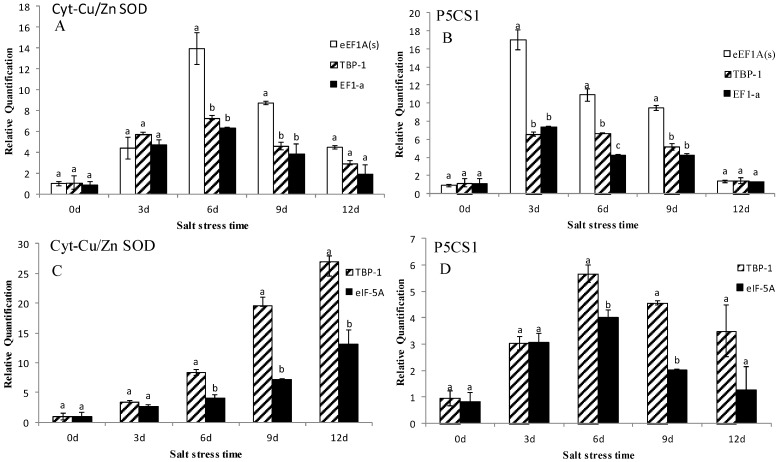
Expression levels of Cyt-Cu/Zn SOD and P5CS1 of annual ryegrass in leaves and roots under salt stress at different times (days 0, 3, 6, 9, 12). (**A**,**B**) represent expression levels of Cyt-Cu/Zn SOD and P5CS1 in leaves. (**C**,**D**) represent expression levels of Cyt-Cu/Zn SOD and P5CS1 in roots. Bars indicate standard error and the different letters above the bars represent significant difference (*p* < 0.05).

In this study, we utilized the 2^−ΔΔCt^ method [38] to calculate the expression of P5CS1 and Cyt-Cu/Zn SOD. As shown in Figure 4, using the most stable gene eEF1A(s), TBP-1 and the least stable gene EF1-a in leaves to normalize the expression, the results suggested that Cyt-Cu/Zn SOD genes are induced with 6- and 7-fold increase on the 3rd day, and a 4- and 5-fold increase on the 6th day. The expression of P5CS1 was induced with a 10- and 9-fold increase on the 3rd day, and a 3, 4- and 5-fold increase on the 6th and 9th day. In roots, using the most stable gene TBP-1 and the least stable gene eIF-5A to normalize the data, the results obviously indicated that expression of Cyt-Cu/Zn SOD and P5CS1 calculated by TBP-1 were higher than those calculated by eIF-5A under salt stress, especially on the 9th day, with both showing 12- and 2-fold increases. In other words, eEF1A(s) is more suitable than TBP-1 and EF1-a in leaves, whereas TBP-1 is more stable than eIF-5A in roots.

### 2.7. Discussion

Annual ryegrass is a grass cultivated worldwide, and with more and more saline soil appearing, its production has been reduced significantly [39]. How to improve the yield of the forage in saline soils has become an important research focus, but the molecular genetic resources of this grass are still underdeveloped and until recently few researchers have analyzed its stably expressed genes. Therefore, to facilitate the understanding of gene expression patterns in this grass, we identified the valid reference genes in leaves and roots under salt stress using qRT-PCR. The accuracy of qRT-PCR method mainly relies on the inclusion of reference genes and preferred method of normalization [40]. There is no reported universal reference gene and there is a fine variation in gene expression in different tissues or under different conditions, so the validation of suitable reference genes in specific tissues or experimental conditions is very necessary. In this work, we selected seven commonly used reference genes to screen for appropriate housekeeping gene for studies on annual ryegrass under salt stress.

There are four popular statistical methods—ΔCt, geNorm, NormFinder and BestKeeper—for calculating the stability of reference gene expression. In the ΔCt method, the stability of genes’ expression it determined by the fluctuation of ΔCt, so the most stable reference gene has the lowest variability in ΔCt values [41], the average expression stability of reference genes was measured by M values in geNorm, and the lower the value, the more stable the reference gene is. Unlike geNorm, in NormFinder the stability (S) values and standard deviation (SD) are utilized to identify the suitable reference genes, and the reference genes were considered stable when they had lower S and SD values. Finally in BestKeeper, correlation coefficients (r values) using produced pair-wise correlations are used to measure the stability, and the higher the r values the higher the SD, and the lower the stability. The results of the four methods are different, probably because they have different statistical algorithms, and there is no consensus on what method should be used, although some reports have showed that the geNorm is the best among them [42]. To obtain a better and more comprehensive evaluation, here we used a new method—RefFinder—to measure the stability of the reference genes under salt stress in different tissues, which is based on software and the four recommended methods and is currently the major statistical algorithm for this purpose. 

Our RefFinder analysis indicated that eEF1A(s) was the most stable gene in leaves under salt stress (Table 2). This has also been identified as the most stable gene in perennial ryegrass (under different defoliation management regimes) [11], black gram (under salt stress) [43] and rice (in different tissues and developmental stages) [44]. In perennial ryegrass, eEF1A(s) has further been used to normalize the gene expression under salt stress [23,37], but interestingly, Xia *et al*., reported eEF1A was the least stable gene in African oil palm [45]. There are lots of novel reports claiming that EF1-a, was the best reference gene in different tissues and different experimental conditions in cotton [46], wheat [47], wheat [48] and cucumber [49]. However, in this study EF1-a was the least stable in leaves (Table 2), which is in agreement with results in eggplant [40] and *Anthurium andraeanum* (Hort.) [50]. Moreover, TBP-1 was the most stable reference gene in roots in all samples of this study (Table 3 and Table 4), which is also similar to perennial ryegrass [12]. In contrast, eIF-5A was the least stable gene (Table 3 and Table 4). Wang *et al*., concluded that eIF-5A is an unstable reference gene for cotton under salt stress [46], whereas eIF-5A was considered valid under abiotic and biotic stress in banana [5]. In a word, there are no ideal internal reference genes, and the expression of the reference genes was not stable in different tissues or under different experimental conditions. In spite of the different concentrations of the same stressor, the suitable reference genes were different [46]. In order to solve this problem, recent reports indicate that choosing more than one gene for normalization could avoid relatively large errors, therefore, a combination of three or more reference genes to normalize the target gene was reliable [51,52,53], but experiments with more internal genes would be more complex and expensive than those using a specific single reference gene for particular conditions. Nowadays an increasing number of studies suggest that the suitable reference genes are specific to different conditions, so for each experimental scenario, we should choose a single reference gene for use in different stages or different tissues [54,55,56,57]. Thus in salt stress-related studies of annual ryegrass, we should choose eEF1A(s) to normalize the leaves data, and use TBP-1 to calculate the expression of the target genes in roots.

## 3. Experimental Section

### 3.1. Plant Materials and Growth Conditions

Two annual ryegrass cultivars were used as plant material, of which “Tetragold” is a tetraploid cultivar released by Barenbrug Co. (Beijing, China), and “R102-3” is a new strain provided by Sichuan Agriculture University (Chengdu, China). The seeds were sown in sand-cultures placed in a growth chamber under a 8 h photoperiod at temperatures of 25 °C (day) and 15 °C (night). The photosynthetically active radiation was 100 μmol m^−2^s^−1^. The seedlings were irrigated with 1/2 strength Hoagland’s solution after germination. When the plants had 3–4 leaves, salt treatment began.

### 3.2. Treatments

Seedlings were subjected to two salinity levels (0 and 300 mM NaCl). Salinity treatments were accomplished by adding 0 (control) and 300 mM NaCl to the 1/2 strength Hoagland’s solution. Roots and leaves were harvested at 0, 3, 6, 9, 12 d after treatment for expression analysis. All treatments and the two genotypes were designed in a randomized, complete block design with three replicates.

### 3.3. RNA Isolation and cDNA Synthesis

Total RNA was isolated from leaves and roots using a RNAsimple Total RNA Kit (TianGen Biotech Co., Ltd., Beijing, China) according to the manufacturer’s instructions. Then it was quantified by the NanoDrop ND-1000 Spectrophotometer (NanoDrop Technologies, Wilmington, DE, USA) at 260/280 nm ratios and 260/230 nm. The RNA quality was confirmed on 1% agarose gel. The cDNA was synthesized from 0.8 μg total RNA using reverse transcription with an iScript cDNA Synthesis Kit (Bio-Rad Laboratories Inc., Hercules, CA, USA) according to the manufacturer’s instructions, and the concentration of each sample was diluted to the same level with nuclease-free water stored at −80 °C.

### 3.4. Primer Design and qRT-PCR Analysis

The annual ryegrass nucleotide sequence and EST were obtained from the GenBank database (NCBI). For EF1-a gene’s available expressed sequence tags we used an orthologous designed gene of Hordeum vulgare; for the eEF1A (s), TBP-1 and YT521-B genes orthologous genes of perennial ryegrass were used; the Cyt-Cu/Zn SOD and P5CS1 genes used were from *Triticum aestivum* and *Deschampsia antarctica*, respectively. We used these gene sequences to clone and sequence to confirm the PCR products. The similarity level between annual ryegrass and the other orthologous genes were 88%–98%. Finally we designed the primers through annual ryegrass sequences, and all primers were designed using Primer Premier 5.0, which were identified by Blastn at NCBI.

qRT-PCR reactions were performed using the Applied Biosystems 7500/7500 fast Real-time PCR system (Bio-Rad). Each reaction contains 10 μL 2 × F S Universal SYBR Green Master supermix (Roche, Shanghai, China), 3 μL (20 ng/μL) diluted cDNA, 4 μL dH_2_O and 1.5 μL (5 pmol/μL) for each primer in a total volume of 20 μL. We chose the annealing temperature using gradient PCR, and the best stability reaction cycling conditions were: 5 min for 95 °C, 40 cycles of 95 °C for 30 s, 56 °C for 30 s, and 72 °C for 30 s. Three technical replicates were analyzed for each biological replicate. Finally, the cycle threshold (C_T_) values were determined for analysis.

### 3.5. Data Analysis

We used delta C_T_ method, geNorm (v 3.5) [5,58] (Vandesompele Jo; Ghent, Belgium), BestKeeper (v 1.0) [8] (Pfaffl Michael W., Munich, Germany) and Normfinder, (v 0.953) [54] (Andersen Claus Lindbjerg, Aarhus, Denmark)software to analyze gene expression stability. The algorithms of them were different in order to the rank can’t be coincident [49], but the RefFinder [59] could provide overall final ranking through calculate the geometric mean of individual gene’s appropriate weight. Use the rank of number one and the last one to calculate the P5CS1 and Cyt-Cu/Zn SOD gene’s expression to identify the effect of the reference genes.

## 4. Conclusions

In conclusion, we screened seven candidate reference genes and identified eEF1A(s) and TBP-1 as most stable ones in leaves and roots of annual ryegrass, respectively. eEF1A(s) as internal reference gene in leaves was overall more reliable than TBP-1 for all samples. The reference gene selected in this investigation will be utilized for evaluation of target gene transcript levels under salt stress in leaves, which could provide a basis for understanding the mechanisms of salt tolerance.
